# Integrated Metagenomic and Metabolomic Analyses Reveal a Microbiota–Metabolite Axis Associated with Gallstone Pathogenesis

**DOI:** 10.3390/metabo15110714

**Published:** 2025-10-31

**Authors:** He Bai, Kai Luo, Yuzhu Jin, Xu Sun, Xudong Zhang, Yuting Zhao, Yaqoob Muhammad, Anliang Huang, Peiyuan Yin, Guixin Zhang

**Affiliations:** 1 Department of General Surgery, The Second Hospital of Dalian Medical University, Dalian 116000, China; baih01@dmu.edu.cn (H.B.); luok@dmu.edu.cn (K.L.); jiangxt@dmu.edu.cn (Y.J.); sunx08@dmu.edu.cn (X.S.); zhangxd02@dmu.edu.cn (X.Z.); wenj20@dmu.edu.com (Y.M.); 2 Laboratory of Integrative Medicine, The First Affiliated Hospital of Dalian Medical University, Dalian 116000, China; zhaoyt03@dmu.edu.cn (Y.Z.); huangal@dmu.edu.cn (A.H.); 3 Institute (College) of Integrative Medicine, Dalian Medical University, Dalian 116000, China

**Keywords:** gallstone disease, dietary inflammatory index, microbiota–metabolite axis, glycodeoxycholate, N-acetylarginine

## Abstract

Background/Objectives: Gallstone disease is a prevalent digestive disorder worldwide, with incidence increasingly linked to obesity, insulin resistance, and dyslipidemia. Emerging evidence suggests that intestinal microbial communities and their bioactive compounds play a central role in pathogenesis. Here, we aimed to clarify whether diet-related microbial alterations and circulating metabolites contribute to gallstone formation. Methods: We integrated dietary inflammatory index (DII) evaluation, genetic analyses of large-scale cohorts, and a cholesterol gallstone mouse model induced by a lithogenic diet (LD). Serum and fecal samples were subjected to metabolomic and metagenomic profiling, followed by multi-omics integration to identify links between microbial taxa, metabolites, and gallstone risk. Results: Higher DII scores were associated with increased gallstone risk. Genetic evidence supported bile acid and amino acid metabolism as potential mediating pathways, with *Akkermansia muciniphila* linked to decreased N-acetylarginine levels and *CAG-448* showing an inverse association with glycodeoxycholate (GDCA). In LD-fed mice, shotgun metagenomics revealed enrichment of lithogenic taxa such as *Bacteroides stercorirosoris* and *Enterocloster*, whereas protective taxa, including *Akkermansia muciniphila* and *CAG-448,* were markedly depleted. Untargeted metabolomics confirmed elevations of GDCA and N-acetylarginine together with broader bile acid imbalance, amino acid stress, and long-chain acylcarnitine accumulation. Correlation analyses further showed that protective taxa were inversely associated with risk metabolites, whereas gallstone-enriched microbes displayed the opposite pattern. Conclusions: This study provides evidence consistent with a contributory role of gut microbiota–metabolite dysregulation in gallstone pathogenesis. Specific taxa (*A. muciniphila*, *CAG-448*) and metabolites (GDCA, N-acetylarginine) may serve as potential biomarkers or targets for microbiota- and diet-based prevention strategies.

## 1. Introduction

Gallstone disease, or cholelithiasis, is one of the most common digestive disorders worldwide [1]. It exerts considerable pressure on medical systems, leading to large numbers of cholecystectomy procedures every year in both the United Kingdom and the United States [2]. Beyond its direct digestive consequences, gallstone disease is associated with various systemic complications, highlighting its wide-ranging impact on human health and quality of life [3,4].

Dietary patterns play an important role in gallstone risk. The Dietary Inflammatory Index (DII), a scientifically validated measure, quantifies the inflammatory potential of an individual’s diet by integrating the intake of various nutrients with either pro- or anti-inflammatory properties [5,6]. Consistent with previous findings, an incremental rise in the DII has been linked to a proportional increase in gallstone susceptibility, with the effect being more pronounced in individuals with obesity [7]. Differences between inflammatory and antioxidant dietary patterns shape the metabolic milieu, with pro-inflammatory diets predisposing individuals to an environment that promotes gallstone formation.

The etiology of gallstone disease is multifactorial. Established risk factors include female sex, advanced age, excessive body weight, insulin-resistant diabetes mellitus, hepatic steatosis, and rapid weight loss [8]. Beyond these confirmed contributing factors, the gastrointestinal microbiome has been recognized as a vital coordinator of metabolic processes and inflammatory equilibrium [9,10]. The gut microbiota, consisting of a complex and diverse array of bacterial species, plays a pivotal role in regulating bile acid metabolism and determining bile acid composition [11,12]. Dysbiosis has been implicated in gallstone formation through mechanisms such as accelerating cholesterol crystallization and inducing chronic inflammation, and generating reactive oxygen species (ROS) [13].

Microbial metabolites serve as critical functional mediators between the gut microbiota and host physiology. Consequently, systematic profiling of the host metabolome is crucial for elucidating the mechanisms by which microbial activities impact health. Altered bile acid homeostasis and systemic metabolite profiles, including bile acid derivatives and lipid intermediates, are strongly associated with gallstone progression [14,15]. For example, *Desulfovibrionales* species enhance bile acid and cholesterol metabolism, favoring lithogenesis [16], whereas *Lactobacillus reuteri* and *Lactobacillus plantarum* alleviate dysbiosis-induced metabolic disturbance through restoring bile acid homeostasis by regulating bile salt hydrolase and FXR-related signaling [17]. The cholesterol gallstone mouse model induced by an LD is widely used to model the metabolic and microbial disturbances of human gallstone pathogenesis [18]. Notably, this model has shown that microbiota dysbiosis may precede and contribute to the disruption of bile acid and cholesterol metabolism during lithogenesis [19].

Despite these advances, the precise relationships among gut microbiota, metabolites, and gallstone disease remain incompletely understood, primarily stemming from analytical complications such as confounder control and temporal relationship uncertainties in cross-sectional research [15]. To address this knowledge gap, our study was designed to integrate genetic epidemiology with experimental validation. We hypothesized that a multi-omics approach, combining deep metagenomic and metabolomic profiling within a controlled LD-induced mouse model, would allow us to substantiate relationships suggested by large-scale human data. This integrative “bench-to-population” strategy aims to refine mechanistic insight into the microbiota–metabolite axis and inform potential microbiota-targeted interventions.

## 2. Materials and Methods

### 2.1. Global Burden of Disease (GBD) Analysis

This study analyzed global cholelithiasis burden using the 2021 Global Burden of Disease (GBD) dataset accessed via the Global Health Data Exchange (GHDx). We stratified 204 countries/regions by age-standardized incidence rates in 1990 and 2021, selecting the top 40 and bottom 40 nations annually to identify 75 overlapping high/low-incidence countries. To enhance geographic and socioeconomic representation, four additional nations (Russia, China, Canada, and the United States) were included, forming a final cohort of 79 countries. For these nations, we extracted Socio-Demographic Index (SDI) data—a composite of education, income, and fertility metrics—and calculated annual percentage changes (AAPC) in incidence from 1990 to 2021 using Joinpoint regression analysis, with asterisks denoting statistically significant trends (α = 0.05). Geospatial trends were visualized through GBD Compare-generated heatmaps, where a blue-to-red gradient depicted escalating years lived with disability (YLDs) across genders and decades, enabling comparative analysis of regional burden dynamics.

### 2.2. Meta-Analysis

The present systematic review followed PRISMA reporting standards. Electronic database searches were conducted across PubMed, MEDLINE, and Embase utilizing the search strategy: (“gallstones” OR “cholelithiasis”) AND (“Dietary Inflammatory Index” OR “DII”). Retrieved information encompassed first author, publication year, study location, research methodology, participant numbers, and adjusted odds ratios (ORs) accompanied by 95% confidence intervals (CIs). When several analytical models were available, we selected the most comprehensively adjusted results. ORs were log-transformed, and standard errors were calculated for subsequent analyses. Inclusion criteria were: (i) studies examining the association between DII and gallstone disease; (ii) reporting ORs or hazard ratios (HRs) with 95% CIs; (iii) observational design (cross-sectional or case–control); and (iv) published in English. Study quality was assessed using the Newcastle–Ottawa Scale (NOS), and only studies scoring ≥ 8 were retained. Based on these criteria, four studies (Luo, Jiang, Wu, and Cheng) were included, whereas two (Ghorbani and Sadri) were excluded.

Statistical analyses were conducted in R (v4.4.2) using the ‘meta’ package (metagen function). A random-effects model was applied to account for both within-study error and between-study heterogeneity, thereby providing robust pooled estimates.

### 2.3. GWAS Design

We applied a two-step Mendelian randomization (MR) framework to explore the potential genetically causal relationships among gut microbiota, circulating metabolites, and the risk of cholelithiasis. MR is a genetic epidemiological approach that uses naturally occurring genetic variants as instrumental variables to infer causality between an exposure and an outcome, thereby minimizing bias from confounding and reverse causation. The validity of MR analysis relies on three core assumptions: (1) Relevance, the selected genetic variants are strongly associated with the exposure of interest; (2) Independence, these variants are not related to other confounding factors; and (3) Exclusion Restriction, they influence the outcome only through the exposure pathway rather than through alternative biological routes [20]. This approach is grounded in Mendel’s law of genetic segregation, which provides a natural form of randomization similar to that used in controlled clinical trials [21].

In the first phase of the analysis, we applied bidirectional MR to explore potential genetic associations between gut microbial taxa and gallstone disease. Genetic variants related to microbial abundance were used as instrumental variables to infer whether genetically predicted alterations in gut microbiota are associated with gallstone risk. Conversely, we examined whether genetic susceptibility to gallstones might correlate with differences in microbial composition. This bidirectional framework helps clarify the predominant direction of genetic associations and minimizes bias from reverse relationships. A similar bidirectional MR analysis was performed between plasma metabolites and gallstone disease.

In the second phase, we conducted a mediation MR analysis to further explore the potential pathways linking gut microbiota to gallstone disease. Based on the findings from Phase 1, candidate microbial taxa and circulating metabolites that showed positive genetic associations with gallstone susceptibility were selected for mediation testing. The analysis aimed to evaluate whether certain metabolites could act as intermediary factors through which genetically predicted microbial variations are associated with gallstone risk. This approach provides a genetic-level perspective on possible microbiome–metabolite–gallstone relationships, while acknowledging that these results indicate statistical associations inferred from genetic instruments rather than direct biological causation. This study was conducted in accordance with the STROBE-MR guidelines [22].

### 2.4. Data Sources for GWAS Exposures and Outcomes

We used summary-level data from large-scale GWAS cohorts. Gut microbiota data for European individuals were obtained from Qin et al., comprising genome-wide genotypes and 16S rRNA profiles of 473 microbial taxa from 5959 participants in the FINNRISK (FR02) cohort [23]. Cholelithiasis data were sourced from the FinnGen R11 database, including 44,582 cases and 397,583 controls of European ancestry [24]. To assess the mediating role of plasma metabolites, we accessed summary GWAS data from Chen et al., based on the Canadian Longitudinal Study on Aging (CLSA), which included 1091 metabolites and 309 ratios measured in 8288 European participants [25]. Dataset details are summarized in Table 1.

### 2.5. Selection of SNPs

Single-nucleotide polymorphisms (SNPs) were selected as instrumental variables (IVs) according to significance thresholds established in previous studies. For gut microbiota, SNPs with *p* < 1 × 10^−5^ were included [26,27]. For plasma metabolites, a threshold of *p* < 5 × 10^−6^ was applied [28,29], and for cholelithiasis, a more strict SNPs (*p* < 5 × 10^−8^) were used [30,31]. To reduce linkage disequilibrium, genetic variants were pruned using the European population data from the 1000 Genomes Project as reference (*R*^2^ < 0.001 within a 10,000 kb window). Ambiguous palindromic SNPs with minor allele frequency (MAF) greater than 0.3 were excluded to avoid strand orientation errors.

The strength of each instrumental variable was evaluated using the F-statistic, calculated as: $F=\frac{R^{2}(N-2)}{1-R^{2}}$, where *R*^2^ represents the proportion of variance in the exposure explained by the SNP, and N is the sample size. *R*^2^ was derived from: $R^{2}=\frac{2\times EAF\times (1-EAF)\times \beta^{2}}{\left(2\times EAF\times \left(1-EAF\right)\times \beta^{2}\right)+(2\times EAF\times (1-EAF)\times {SE}^{2}\times N)}$ [32]. SNPs with F-statistics below 10 were excluded to avoid weak instrument bias [33]. Potential confounding traits were identified from prior literature and included obesity, body mass index (BMI), waist circumference, type 2 diabetes, metabolic syndrome, depression, and lifestyle factors such as smoking, alcohol consumption, and coffee intake [34,35,36]. To ensure that selected SNPs were not strongly related to these confounders, we used the FastTraitR tool to screen for SNP–phenotype associations [37]. Any SNP showing a significant association (*p* < 1 × 10^−5^) with known confounders was removed. This filtering process helped minimize confounding effects and improve the robustness of the Mendelian randomization results.

### 2.6. Mouse Model and Sample Collection

Male C57BL/6J mice (8 weeks old, 20–22 g; Huafukang Bioscience Co., Ltd., Beijing, China) were housed under specific pathogen-free (SPF) conditions with a 12 h light/dark cycle, temperature of 22.5 ± 2 °C, and relative humidity of 55–75%. After 7 days of acclimatization, mice were randomly assigned to two groups (*n* = 12 per group). The control group was maintained on a chow diet (CD; LAD0011, Trophic Animal Feed High-tech Co., Ltd., Nantong, China), while the model group received a lithogenic diet (LD; TP 28900, same manufacturer) for 6 weeks. The LD formulation comprised 15% dairy fat, 1.25% dietary cholesterol, 0.5% cholic acid, 2% vegetable oil, 50% refined sugar, and 20% casein protein, with standard mineral and vitamin supplementation. Gallstone formation was confirmed by examination of gallbladder bile at the endpoint. Ethical approval for all animal procedures was obtained from the Institutional Animal Care and Use Committee of the Dalian Medical University (Approval No. AEE23097). For multi-omics analyses, cecal contents were collected for metagenomic sequencing and serum samples were obtained for untargeted metabolomics.

### 2.7. Gallbladder Contractility Assessment

After a 12 h fast with ad libitum access to water, mice (*n* = 5 per group) were anesthetized with pentobarbital sodium. A midline laparotomy was performed, and a PE-10 polyethylene catheter was inserted into the duodenum, externalized through the left abdominal wall, and connected to an infusion pump. The gallbladder was exposed, and its initial volume was determined by measuring the length (L), width (W), and height (H) with a digital microcaliper and calculating the volume as V = (*π*/6) × L × W × H. Mice then received a 5 min intraduodenal infusion of corn oil (40 μL/min) via the catheter. Ten minutes after completion of the infusion, gallbladder volume was remeasured. The gallbladder emptying rate (%) was calculated as [(initial volume − post-infusion volume)/initial volume] × 100%. All data are presented as mean ± standard deviation (SD). Differences between LD-fed and CD-fed mice were analyzed using an unpaired two-tailed Student’s *t*-test, with *p* < 0.05 considered statistically significant.

### 2.8. Bile Collection and Biochemical Analysis

Mice were anesthetized with pentobarbital, and the abdomen was opened under sterile conditions. For gallbladder bile collection, the common bile duct was ligated to block bile flow, and the gallbladder was gently emptied using a syringe fitted with a 31-gauge needle. All bile samples were immediately placed on ice and stored at −80 °C for further analysis. Cholesterol and bile acid concentrations were determined using commercial assay kits (Elabscience Biotechnology Co., Ltd., Wuhan, China), while phospholipid levels were measured using phospholipid detection kits (Jiangsu Edison Biotechnology Co., Ltd., Yancheng, China). All assays were performed according to the manufacturers’ protocols. The cholesterol saturation index (CSI) was calculated according to the Carey and Small table method [38]. Bile biochemical parameters were measured in mice (*n* = 5 per group), and group differences between LD-fed and CD-fed mice were analyzed using an unpaired two-tailed Student’s *t*-test, with *p* < 0.05 considered statistically significant.

### 2.9. Metagenomic Sequencing

Total DNA was extracted from fecal samples obtained from LD–fed and CD–fed mice (*n* = 8 per group) using the Genome DNA Extraction Kit (TIANGEN, Beijing, China) according to the manufacturer’s instructions. DNA libraries were constructed using the TruSeq Nano DNA Library Preparation Kit-Set A (#FC-121–4001, Illumina, San Diego, CA, USA) following the manufacturer’s protocol. Metagenomic libraries were sequenced on an Illumina NovaSeq 6000 platform (paired-end 150 bp, PE150) at LC-Bio Technology Co., Ltd. (Hangzhou, China).

Fastp (v0.23.4) was used to remove reads containing adapter contamination, low-quality bases, or undetermined bases, and to verify overall sequence quality. Quality-filtered reads were aligned to the host genome (Mus musculus) using Bowtie2 (v2.5.1) to remove host-derived sequences. The remaining reads were de novo assembled for each sample using MEGAHIT (v1.2.9) and subsequently used for microbial taxonomic and functional annotation. MetaGeneMark (v3.26) was employed to predict coding sequences (CDSs) from the assembled contigs, and CDSs from all samples were clustered using MMseqs2 (v15-6f452) to obtain unigenes. DIAMOND (v0.9.14) was used to perform taxonomic classification of the microbiota against the NCBI NR database, and microbial functions were annotated with reference to the Kyoto Encyclopedia of Genes and Genomes (KEGG) database.

### 2.10. Untargeted Metabolomics

Serum metabolomic analysis was performed on samples collected from LD–fed and CD–fed mice (*n* = 8 per group) using a Thermo Scientific Ultimate™ 3000 UHPLC system connected to a Q Exactive™ Plus Orbitrap mass spectrometer (Thermo Fisher Scientific, Waltham, MA, USA). Chromatographic separation was conducted in both positive and negative ion modes. For cationic detection, samples underwent fractionation on an Acquity™ HSS T3 column (2.1 × 100 mm, 1.8 µm; Waters, Milford, MA, USA) utilizing a stepped gradient comprising water (0.1% formic acid) and acetonitrile (0.1% formic acid). For anionic analysis, compound resolution was accomplished using an Acquity™ BEH C18 column (2.1 × 100 mm, 1.7 µm; Waters, Milford, MA, USA) with ammonium bicarbonate–containing eluents. Mobile phase delivery occurred at 0.4 mL/min while column heating was maintained at 50 °C. Mass spectrometry was carried out in heated electrospray ionization (HESI) mode. Full-scan spectra were acquired at 70,000 resolution (FWHM) across 70–1000 *m*/*z*, with an AGC target of 1 × 10^6^ and maximum injection time of 200 ms. To monitor instrument stability and facilitate metabolite identification, pooled QC samples were injected periodically and analyzed in data-dependent acquisition (DDA) mode (TopN = 10) at 17,500 resolution.

Primary datasets were processed in TraceFinder™ (Thermo Fisher Scientific) for peak detection, retention time alignment, and feature integration. Metabolites were identified through precise mass determination, retention time, and MS/MS spectra, matched against HMDB, METLIN, and an in-house standard library.

### 2.11. Data Analysis

#### 2.11.1. Microbiome and Metabolomics Data Analysis

Microbiome analyses were performed in QIIME2, including alpha diversity (Observed species, Chao1, Shannon, Simpson, Pielou) and beta diversity (Bray–Curtis dissimilarity, PCoA). Group differences were tested using the Wilcoxon rank-sum test (alpha diversity) and PERMANOVA (beta diversity). Differentially abundant taxa were identified with LEfSe (LDA score > 2.0, *p* < 0.05).

Metabolomics data were log10-transformed prior to analysis. Weighted gene co-expression network analysis (WGCNA) was applied to identify metabolite co-abundance modules. Modules were merged when eigengene correlations exceeded 0.85. Associations between modules, specific metabolites, and microbial taxa were assessed by Spearman correlation, with multiple testing corrected by the Benjamini–Hochberg method.

#### 2.11.2. Mendelian Randomization Analysis

All MR analyses were performed in R (version 4.4.1) using the TwoSampleMR (version 0.5.7) and MendelianRandomization (version 0.7.0) packages. The primary analytical approach was inverse variance weighting (IVW), which provides an overall estimate by combining the effects of individual genetic instruments. To ensure robustness, complementary methods including the weighted median (WM) and mode-based estimators were also applied. Statistical significance was set at *p* < 0.05.

Instrument heterogeneity, which reflects variability in SNP-specific causal estimates, was evaluated using Cochran’s Q statistic [39], Excessive heterogeneity may indicate that some variants influence the outcome through mechanisms unrelated to the exposure. Horizontal pleiotropy, another potential source of bias, occurs when a genetic variant affects the outcome through alternative biological pathways independent of the exposure. To detect and adjust for these effects, MR-Egger regression and MR-PRESSO were applied [40]. A genetic association was regarded as reliable when the results were consistent across multiple MR methods (IVW, WM, MR-Egger and MR-PRESSO) and remained significant in the IVW mode [41,42].

For mediation MR analyses, the total association between gut microbiota and cholelithiasis (β_all) was decomposed into direct and indirect components. The indirect (mediated) effect was estimated as β_1_ × β_2_, representing the genetic pathway from microbiota to metabolite to cholelithiasis, while the direct effect was calculated as β_all − (β_1_ × β_2_). The proportion of mediation was then determined as (β_1_ × β_2_)/β_all. This framework enabled an assessment of potential metabolic mediation at the genetic level, while acknowledging that MR reflects genetic associations rather than definitive biological causation.

## 3. Results

### 3.1. Global Prevalence and Regional Distribution of Cholelithiasis

**Figure 1:**
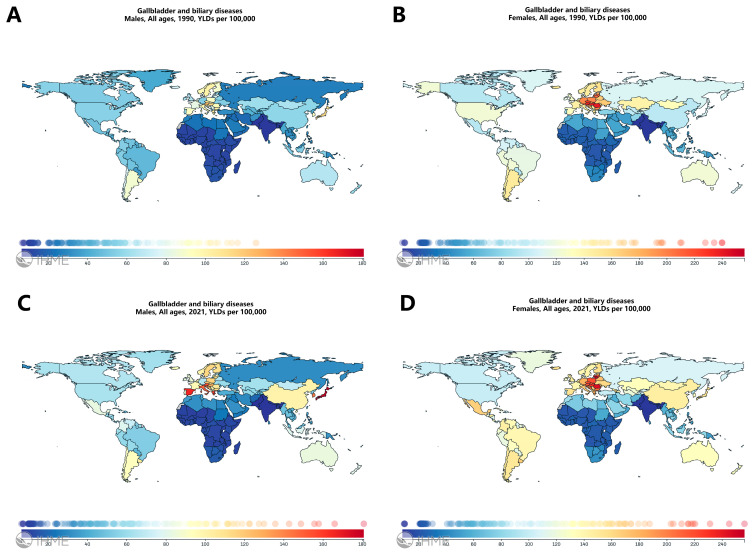
Distribution of YLDs due to gallbladder and biliary diseases in men and women worldwide, 1990 and 2021. (**A**) Age-standardized Years Lived with Disability (YLDs) for cholelithiasis in males, 1990. (**B**) Males, 2021. (**C**) Females, 1990. (**D**) Females, 2021. World maps were generated using the Global Burden of Disease Compare Tool. The color gradient from blue to red indicates disability-burden intensity, with darker red representing higher YLDs per 100,000 population.

GBD analyses revealed a marked global rise in years lived with disability (YLDs) due to cholelithiasis between 1990 and 2021 (Figure 1A–D). The upward trend was consistent across both sexes, indicating that the disease burden has increased universally.

Regional heterogeneity was evident: economically developed regions such as Europe maintained high YLD levels throughout the observation period, while Africa and South Asia remained at the lower end. East Asia and South America showed the most rapid growth, with YLDs increasing by over 40% since 1990. In North America, men exhibited relatively stable YLDs, whereas women showed a decline (Supplementary Table S1). These geographic differences likely reflect interactions between socioeconomic status, dietary transitions, and healthcare accessibility.

### 3.2. Dietary Inflammatory Index (DII) and Gallstone Risk

**Figure 2:**
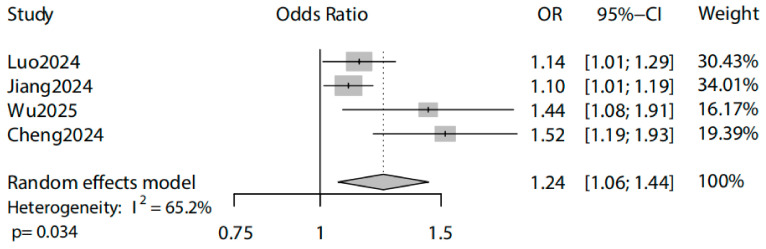
Association between Dietary Inflammatory Index (DII) and risk of cholelithiasis. Forest plot of a random-effects meta-analysis including four U.S.-based studies (2024–2025; *n* = 36,662). Each square represents the odds ratio (OR) for gallstone risk per unit increase in the DII, with horizontal lines showing 95% confidence intervals (CI). The diamond indicates the pooled effect estimate. A higher DII was significantly associated with increased risk of cholelithiasis (pooled OR = 1.24, 95% CI: 1.06–1.44), despite moderate heterogeneity across studies (*I^2^* = 65.2%, *p* = 0.034). Statistical analysis was performed using a random-effects meta-analysis model; *p* < 0.05 was considered statistically significant. The data were obtained from Luo et al. [43], Jiang et al. [44], Wu et al. [7], and Cheng et al. [45].

The meta-analysis included four U.S.-based studies published between 2024 and 2025, encompassing 36,662 participants (sample sizes 6623–12,426; Table 2). A higher DII was significantly associated with an increased risk of cholelithiasis, with a pooled odds ratio of 1.24 (95% CI: 1.06–1.44) under a random-effects model (Figure 2). Despite moderate heterogeneity (*I^2^* = 65.2%, *p* = 0.034), the consistent direction of effect across studies supports a robust association between a pro-inflammatory diet and gallstone disease.

### 3.3. Mendelian Randomization Analysis of Gut Microbiota and Cholelithiasis

**Figure 3:**
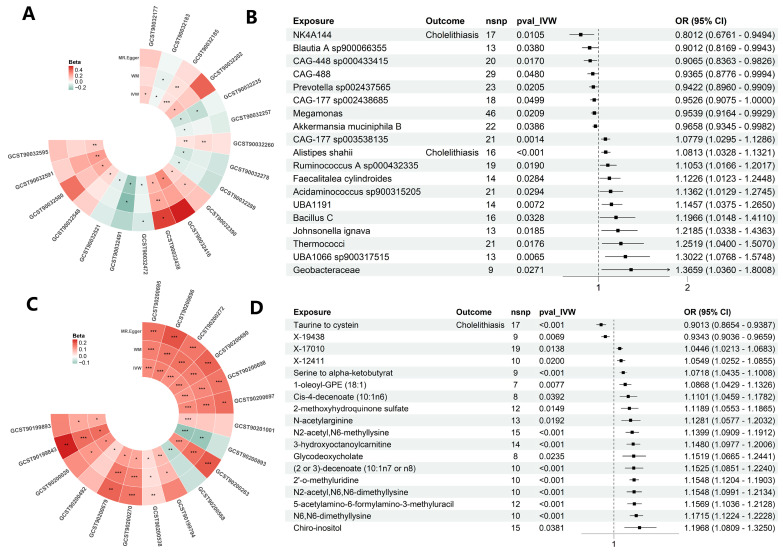
Causal effects of the gut microbiota and plasma metabolites on cholelithiasis. (**A**) Circular Manhattan plot showing microbial taxa tested by Mendelian randomization (MR). Taxa highlighted represent those with significant causal effects on cholelithiasis. (**B**) Forest plot of microbial taxa with significant effects. A total of 19 taxa were identified, with 11 conferring increased risk and 8 showing protective effects. Notably, *Akkermansia muciniphila* and *CAG-448* were associated with reduced gallstone risk. Odds ratios (ORs) and 95% confidence intervals (CIs) are shown. (**C**) Circular Manhattan plot screening plasma metabolites for causal associations with cholelithiasis. (**D**) Forest plot of 18 plasma metabolites identified by MR. Sixteen metabolites increased gallstone risk, while two were protective (e.g., taurine to cysteine ratio). Statistical analysis was conducted using the inverse variance–weighted (IVW) method within the MR framework; *p* < 0.05 was considered statistically significant (* *p* < 0.05, ** *p* < 0.01, *** *p* < 0.001).

To explore potential associations consistent with causality within the diet–microbiota–gallstone axis, we first performed MR analyses to identify gut taxa potentially linked to cholelithiasis. Many of the identified taxa are known to be sensitive to dietary patterns, supporting their role as mediators. The results suggested that these microbial changes were associated with cholelithiasis risk in a direction consistent with causal inference, thereby providing evidence supportive of a potential diet–microbiota–gallstone axis. Using the inverse variance–weighted (IVW) approach, we identified 19 microbial taxa significantly associated with gallstone risk: 11 taxa were associated with increased risk, whereas 8 were protective (Figure 3A,B). Sensitivity analyses indicated no evidence of horizontal pleiotropy, and heterogeneity was significant only for *Johnsonella ignava* (Supplementary Table S2). Bidirectional MR further excluded reverse causality, as cholelithiasis had no appreciable impact on microbial composition, except for a reduction in *Thermococci* abundance (Supplementary Table S3).

### 3.4. Mendelian Randomization Analysis of Plasma Metabolites and Cholelithiasis

To further investigate the metabolic dimension, MR analysis was performed, identifying 18 plasma metabolites significantly associated with cholelithiasis risk after FDR correction (Figure 3C). Of these, 16 metabolites were linked to an increased risk, while 2 were associated with a reduced risk. Sensitivity analyses revealed no evidence of horizontal pleiotropy (Figure 3D, Supplementary Table S4). Bidirectional MR analysis confirmed that all associations were unidirectional, indicating that cholelithiasis did not exert a reverse effect on the aforementioned plasma metabolites (Supplementary Table S5).

### 3.5. Mediation Analysis of Plasma Metabolites

**Figure 4:**
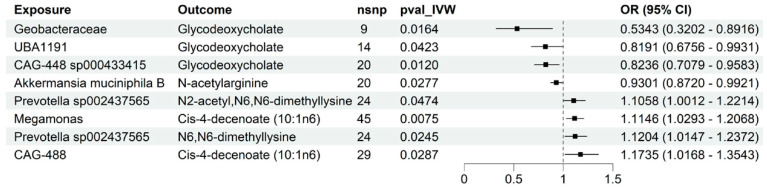
Two-step MR analysis showing associations consistent with causality between gut microbiota and plasma metabolites. Forest plot illustrating associations consistent with causal inference between previously identified gut microbial taxa (*n* = 19) and plasma metabolites (*n* = 18) based on a two-step Mendelian randomization (MR) framework. Eight significant microbiota–metabolite links were identified. Protective taxa such as *Geobacteraceae*, *Akkermansia muciniphila* and *CAG-448* were inversely associated with risk metabolites including N-acetylarginine and glycodeoxycholate (GDCA), whereas taxa such as *Prevotella sp002437565* and *Megamonas* were positively associated with lithogenic metabolites (e.g., cis-4-decenoate). Odds ratios (ORs) and 95% confidence intervals (CIs) are shown. Statistical analysis was conducted using a two-step MR framework applying the inverse variance–weighted (IVW) method; *p* < 0.05 was considered statistically significant.

To further explore whether plasma metabolites may play an intermediary role in the relationship between gut microbiota and cholelithiasis, we conducted a two-step MR framework. In the first step, associations consistent with causality between 19 gut microbial taxa and 18 plasma metabolites were examined, identifying 8 significant microbiota–metabolite pathways (Figure 4, Supplementary Table S6). In the second step, we evaluated the potential intermediary contribution of these metabolites to the microbiota–cholelithiasis association and identified two significant metabolite-associated pathways (Supplementary Table S7). Specifically, GDCA mediated 27.96% of the protective effect of *CAG-448 sp000433415*, N-acetylarginine accounted for 25.10% of the inverse association of *Akkermansia muciniphila B* (Table 3). These results highlight specific plasma metabolites as mechanistic links between gut microbiota and cholelithiasis, consistent with but not definitive of causal pathways underlying disease development.

### 3.6. Validation of the Lithogenic Diet–Induced Gallstone Model

**Figure 5:**
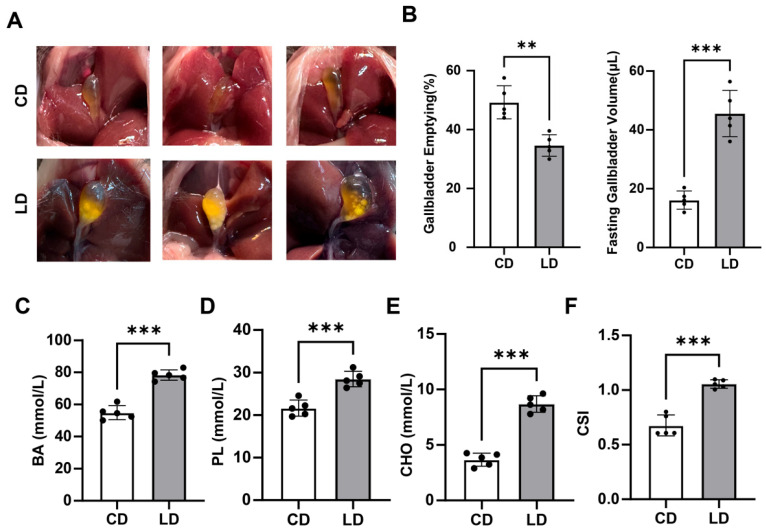
Lithogenic diet–induced gallstone model in mice. (**A**) Representative gross images of gallbladders from mice fed a CD or LD for 6 weeks, showing marked enlargement and yellow, turbid bile with visible cholesterol crystals in LD-fed mice. (**B**) Quantitative analysis showing significantly reduced gallbladder emptying rates and increased fasting gallbladder volumes in LD-fed mice compared with controls. (**C**–**E**) Biochemical assays of gallbladder bile showing elevated concentrations of bile acids (BA), phospholipids (PL), and cholesterol (CHO) in LD-fed mice relative to CD-fed controls. (**F**) The cholesterol saturation index (CSI) was markedly increased in LD-fed mice, indicating bile supersaturation and enhanced lithogenic potential. Data are presented as mean ± SD (*n* = 5 per group). Statistical significance was determined using unpaired two-tailed Student’s *t*-tests; *p* < 0.05 was considered statistically significant (** *p* < 0.01, *** *p* < 0.001). Each black dot represents one mouse (*n* = 5 per group).

To confirm successful establishment of the LD–induced gallstone model, we first evaluated gallbladder morphology, contractility, and bile composition. After 6 weeks of LD feeding, mice exhibited visibly enlarged gallbladders containing yellow, turbid bile, in contrast to the translucent bile observed in CD–fed controls (Figure 5A). Quantitative assessment demonstrated that LD-fed mice showed significantly reduced gallbladder emptying rates and increased fasting gallbladder volumes compared with CD-fed mice (*p* < 0.01 and *p* < 0.001, respectively; *n* = 5 per group, unpaired two-tailed Student’s *t*-test) (Figure 5B, Supplementary Table S8).

Biochemical analysis of gallbladder bile revealed elevated concentrations of bile acids (BA), phospholipids (PL), and cholesterol (CHO) in LD-fed mice (all *p* < 0.001) (Figure 5C–E, Supplementary Table S9). Consistent with these alterations, the cholesterol saturation index (CSI) was markedly increased (*p* < 0.001), indicating bile supersaturation and enhanced lithogenic potential (Figure 5F, Supplementary Table S9). Collectively, these findings confirm that the LD regimen successfully induced a lithogenic state characterized by impaired gallbladder motility and compositional changes conducive to cholesterol crystallization.

### 3.7. Lithogenic Diet Induces Gut Microbiota Dysbiosis in Gallstone Mice

**Figure 6:**
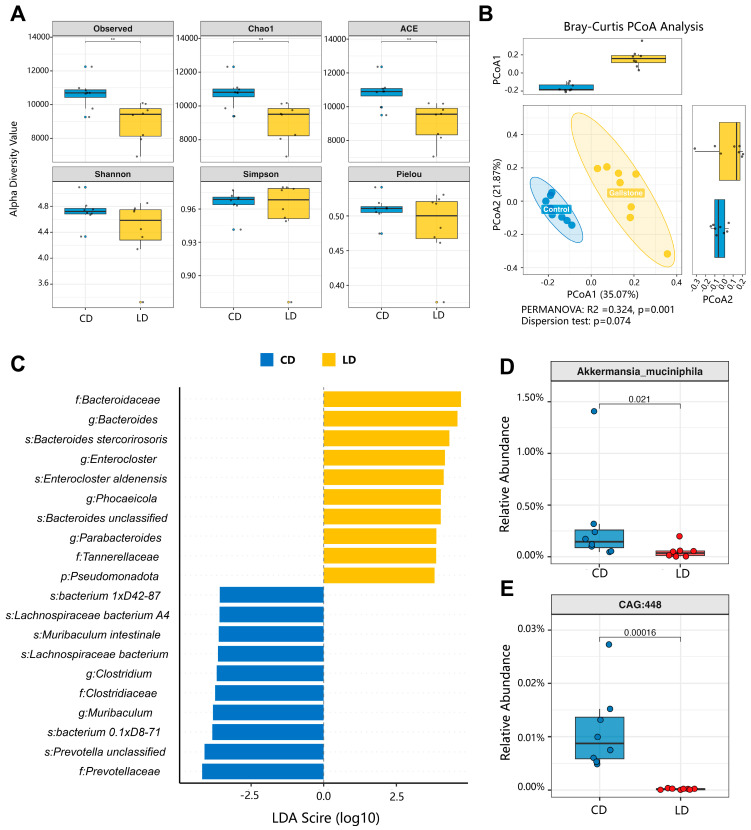
Lithogenic diet induces gut microbiota dysbiosis in gallstone mice. (**A**) α-diversity indices (Observed species, Chao1, ACE, Shannon, Simpson, and Pielou) comparing CD and LD-fed groups, showing significant reductions in species richness but stable evenness (Wilcoxon test, *p* = 0.007 for richness indices). (**B**) β-diversity analysis based on Bray-Curtis distances. Principal coordinate analysis (PCoA) revealed distinct clustering between CD and LD-fed mice, confirmed by PERMANOVA (*R^2^* = 0.324, *p* = 0.001). (**C**) LEfSe analysis (LDA score > 4.0) identified bacterial taxa enriched in the LD group (yellow), including *Bacteroides stercorirosoris* and *Enterocloster aldenensis*, versus taxa enriched in CD group (blue), such as *Prevotellaceae* and *Muribaculaceae*. (**D**,**E**) Relative abundance of protective taxa identified by genetic analyses, *Akkermansia muciniphila* (*p* = 0.021) and *CAG-448* (*p* = 0.00016), which were markedly reduced in LD-fed mice. Data are presented as mean ± SD (*n* = 8 per group). Statistical analyses were performed using Wilcoxon rank-sum tests for α-diversity and PERMANOVA for β-diversity; *p* < 0.05 was considered statistically significant (** *p* < 0.01).

To assess the overall impact of an LD on gut microbiota, we performed shotgun metagenomic sequencing of fecal samples. All sequencing runs showed high-quality metrics with Q30 > 98% and low host DNA contamination (<10%) (Supplementary Table S10). Analysis of α-diversity demonstrated a significant reduction in species richness in gallstone mice, as indicated by Observed species, Chao1, and ACE indices (Wilcoxon test, *p* = 0.007 for all; Figure 6A). In contrast, Shannon and Simpson indices did not differ significantly between groups (*p* = 0.328 and 0.798, respectively), suggesting that community evenness remained stable despite reduced richness. β-diversity analysis based on Bray–Curtis distances revealed clear compositional differences between groups (Figure 6B).

Principal coordinate analysis (PCoA) showed distinct clustering, and PERMANOVA confirmed that group status accounted for 32.4% of the overall variation (*R*^2^ = 0.324, *p* = 0.001). These results indicate that the LD induced pronounced gut microbiota dysbiosis. LEfSe analysis (LDA score > 4.0) further identified key bacterial taxa driving group differences (Figure 6C). Gallstone mice were enriched for multiple members of the phylum Bacteroidota, including *Bacteroides*, *Bacteroides stercorirosoris*, and *Enterocloster*, whereas controls were characterized by higher abundances of *Prevotellaceae* and *Muribaculaceae taxa*.

Beyond these group-level differences, we further examined taxa highlighted by MR analysis. Consistent with the MR findings, two taxa previously identified as being inversely associated with cholelithiasis, *Akkermansia muciniphila* (*p* = 0.021) and *CAG-448* (*p* = 0.00016), were markedly reduced in gallstone mice (Figure 6D–E). These findings provided experimental support for the association patterns suggested by MR analyses and highlight specific microbial alterations observed in the LD–induced murine gallstone model, thereby reinforcing the potential link between gut microbiota dysbiosis and gallstone pathogenesis.

### 3.8. Lithogenic Diet Alters Metabolome in Gallstone Mice

**Figure 7:**
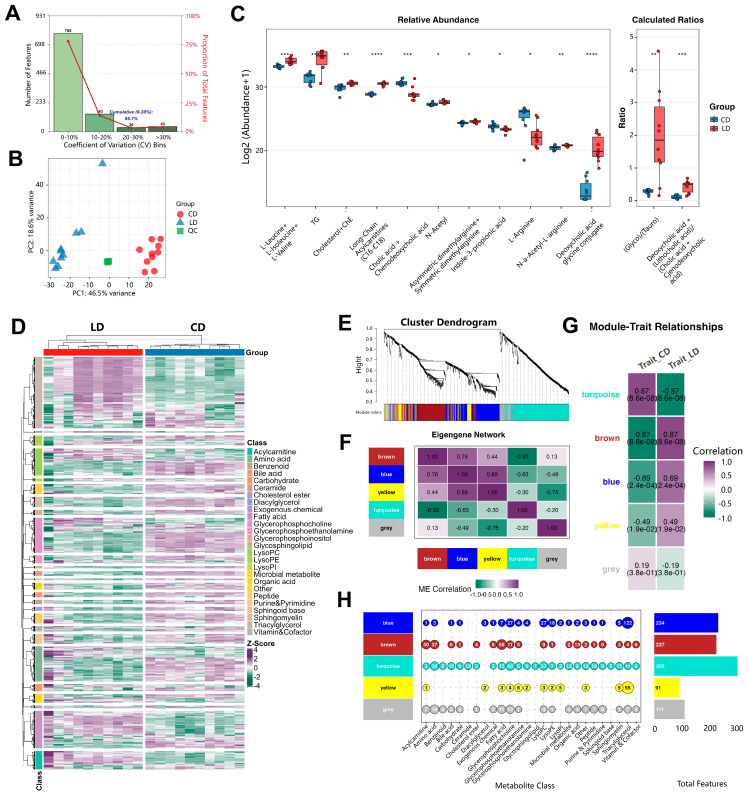
Serum metabolomic alterations in gallstone mice fed a lithogenic diet. (**A**) Coefficient of variation (CV) distribution of detected metabolic features, with >70% falling below the 30% CV threshold, indicating high analytical reproducibility. (**B**) Principal component analysis (PCA) showing clear separation between LD and CD groups. Quality control (QC) injections clustered tightly, confirming data stability and reliability. (**C**) Boxplots of representative differential metabolites and calculated ratios. LD-fed mice exhibited higher serum glycodeoxycholate (GDCA) levels (*p* = 0.029), an increased secondary/primary bile acid ratio (*p* < 0.001), and an elevated glycine/taurine conjugation ratio (*p* = 0.004), as well as increased N-acetylarginine (*p* = 0.003), total N-acetylated amino acids (*p* = 0.017), and branched-chain amino acids (BCAAs) (*p* = 0.002). (**D**) Unsupervised hierarchical clustering heatmap showing widespread abundance shifts across multiple metabolite classes between groups. (**E**) Weighted gene correlation network analysis (WGCNA) dendrogram identifying four major eigengene modules (turquoise, brown, blue, and yellow). (**F**) Module-module correlation matrix. (**G**) Module-trait correlation heatmap showing a strong positive association of the brown module with the gallstone phenotype (*r* = 0.87) and a strong negative association of the turquoise module (*r* = −0.87). (**H**) Module-metabolite class enrichment analysis. The brown module was enriched for fatty acids and acylcarnitines, while the turquoise module was enriched for phospholipids and bile acid-related species. Data are presented as mean ± SD (*n* = 8 per group). Statistical analyses were performed using the Wilcoxon rank-sum test for metabolite comparisons, with *p* < 0.05 considered statistically significant (* *p* < 0.05, ** *p* < 0.01, *** *p* < 0.001, **** *p* < 0.0001).

To further assess the impact of the LD on host metabolism, we performed untargeted serum metabolomics. QC injections clustered tightly in the PCA space, and most detected features fell below the 30% CV threshold (Figure 7A). Moreover, PCA showed distinct clustering of gallstone and control groups (Figure 7B), indicating that the data were robust and representative.

Consistent with MR-informed pathways, bile acid metabolism was perturbed. Serum GDCA was significantly elevated in gallstone mice (*p* = 0.029), and calculated ratios-including the secondary/primary bile acid ratio (*p* < 0.001) and the glycine/taurine conjugation ratio (*p* = 0.004)-were increased (Figure 7C), suggesting enhanced microbiota-mediated bile acid transformation and greater lithogenic potential. In addition, amino acid metabolism was perturbed. N-acetylarginine was significantly higher in gallstone mice (*p* = 0.003), accompanied by increases in the total pool of N-acetylated amino acids (*p* = 0.017) and branched-chain amino acids (BCAAs; *p* = 0.002) (Figure 7C), pointing to broad disturbances in amino-acid homeostasis.

At the feature/class level, an unsupervised heatmap revealed widespread abundance shifts across multiple metabolite classes (Figure 7D), indicating broad metabolic remodeling beyond bile acid and amino acid pathways. To capture coordinated changes, we applied weighted correlation network analysis (WGCNA). Module detection identified four major eigengene modules (turquoise, brown, blue, yellow; Figure 7E). Module–module correlations are shown in Figure 7F. Module-trait correlations demonstrated a strong positive association of the brown module with the gallstone phenotype (*r* = 0.87) and a strong negative association of the turquoise module (*r* = −0.87; Figure 7G). Class-enrichment profiling revealed that the brown module was enriched for fatty acids and acylcarnitines, whereas the turquoise module was enriched for phospholipids and bile-acid-related species (Figure 7H). Accumulation of long-chain acylcarnitines within the brown module points to impaired mitochondrial β-oxidation, suggesting a potential metabolic hub linking upstream risk factors to downstream gallstone pathology.

### 3.9. Integrated Analysis Reveals Functional Associations Between Microbiota Dysbiosis and Metabolic Networks

**Figure 8:**
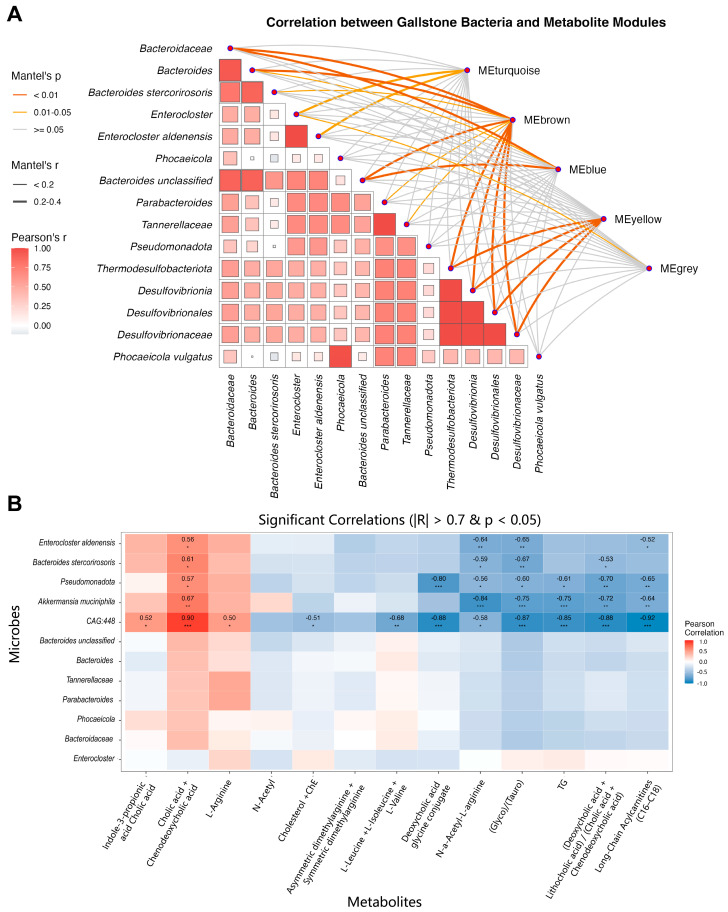
Integrated analysis of gut microbiota and serum metabolome in gallstone mice. (**A**) Mantel test analysis showing associations between LEfSe-identified microbial taxa and WGCNA-derived metabolite modules. Edge colors indicate significance levels, and line thickness reflects correlation strength. The heatmap in the lower-left panel represents Pearson correlation coefficients among microbial taxa. Significant module–microbiota correlations were determined by Mantel’s test (*r* = 0.227–0.316, *p* < 0.05). (**B**) Pearson correlation heatmap displaying associations between key microbial taxa and representative metabolites, filtered by |*r*| > 0.7 and *p* < 0.05 (two-tailed). Statistical significance was determined using Mantel’s test and Pearson correlation analysis (*n* = 8 per group), with *p* < 0.05 considered significant (* *p* < 0.05, ** *p* < 0.01, *** *p* < 0.001).

To explore functional connections between gut microbial dysbiosis and host metabolic remodeling, we performed Mantel tests integrating LEfSe-identified microbial biomarkers with WGCNA-derived metabolite modules. Significant associations were observed between multiple metabolite modules and microbial community structures. The brown module, previously linked to mitochondrial dysfunction, showed the strongest correlation (Mantel’s *r* = 0.316, *p* = 0.001), followed by the yellow (Mantel’s *r* = 0.245, *p* = 0.001) and blue modules (Mantel’s *r* = 0.242, *p* = 0.009). The turquoise module, reflecting depletion of protective metabolites, also displayed significant correlations (Mantel’s *r* = 0.227, *p* = 0.023) (Figure 8A).

At the individual taxon-metabolite level, Pearson correlation analysis further supported MR-predicted associations. The protective taxon *CAG-448* showed positive correlations with primary bile acids such as cholic acid (*r* = 0.90, *p* < 0.001) and strong negative correlations with lithogenic secondary bile acids including deoxycholic acid (*r* = −0.88, *p* < 0.001) and its conjugated forms (*r* ≤ −0.87, *p* < 0.001). Similarly, *Akkermansia muciniphila* abundance was inversely correlated with N-acetylarginine (*r* = −0.84, *p* < 0.001), showing a pattern consistent with the associations predicted by MR (Figure 8B).

Both *CAG-448* and *A. muciniphila* were negatively correlated with triglycerides and long-chain acylcarnitines, whereas gallstone-enriched taxa such as *Bacteroides stercorirosoris* and *Enterocloster aldenensis* showed the opposite associations. Together, these findings demonstrate functional coordination between microbial dysbiosis and host metabolic networks, consistent with a connected diet–microbiota–metabolite–gallstone axis in the murine model.

## 4. Discussion

Gallstone disease is a common digestive disorder with a rising global burden, largely driven by metabolic disturbances such as obesity, insulin resistance, and dyslipidemia [16]. These features highlight its close connection to systemic cholesterol, bile acid, and energy metabolism rather than a purely local biliary process. In this context, our study provides integrative evidence that pro-inflammatory dietary patterns promote gallstone disease through gut microbiota–mediated metabolic reprogramming.

At the epidemiological level, consistent with previous studies, we observed pronounced heterogeneity in gallstone burden across populations [46]. Europe and East Asia showed high incidence and rapid growth, while South Asia and Africa were less affected. Even high-income countries such as the United States and Canada displayed rising incidence, reflecting the impact of westernized lifestyles. Women consistently exhibited higher incidence and disability burden than men, likely due to estrogen-mediated modulation of hepatic cholesterol metabolism [47]. Together, these observations highlight the interplay between environmental transitions and biological factors in gallstone pathogenesis. The role of diet is further supported by the DII, as pro-inflammatory dietary patterns rich in fat and poor in fiber promote gut dysbiosis, reduce short-chain fatty acid production, and perturb bile acid homeostasis [48]. This diet–microbiota–inflammation axis suggests a potential link between epidemiological trends and host metabolic disruption, prompting our MR analyses to explore associations consistent with causal inference.

Our MR analyses highlighted multiple metabolic axes through which gut microbiota influence gallstone risk, including bile acid derivatives (e.g., GDCA), amino acid intermediates (e.g., N-acetylarginine), and lipid species (e.g., cis-4-decenoate). This pattern suggests that gallstone pathogenesis is characterized by broad metabolic disturbances rather than a single dysregulated pathway. Within this landscape, amino acid and bile acid metabolism emerged as central mediators. *Akkermansia muciniphila* appeared as a protective taxon, reducing circulating N-acetylarginine, a metabolite involved in nitric oxide signaling and cardiovascular regulation [49]. This aligns with prior evidence of *A. muciniphila* in maintaining metabolic homeostasis and extending its potential benefits to biliary physiology, supporting its candidacy as a probiotic for gallstone prevention.

By contrast, *CAG-448* exerted a predominantly protective effect by decreasing GDCA, a bile salt whose excess impairs cholesterol solubilization via micelle formation [50]. In addition, *CAG-448* was positively correlated with protective primary bile acids and inversely associated with lithogenic secondary bile acids, reinforcing its protective role. Although minor associations with certain lipid intermediates were observed, these appear secondary and do not outweigh its overall protective impact. Overall, the dominant impact of *CAG-448* appears protective, though secondary pathways may introduce complexity. These findings emphasize the need to move beyond taxon-level associations toward functional characterization of microbial pathways. In this regard, our LD–induced mouse experiments provided complementary validation by confirming metabolite shifts consistent with MR predictions, thereby supporting evidence consistent with a causal relationship for candidate taxa such as *CAG-448*.

Experimental validation in LD–induced mice further supported these MR-based inferences. Among the altered metabolites, GDCA and N-acetylarginine showed consistent elevations with MR predictions, suggesting their cross-species relevance. GDCA accumulation reflects enhanced microbial 7α-dehydroxylation and a shift toward glycine-conjugated bile acids, changes that increase bile hydrophobicity, reduce cholesterol solubility, and promote cholesterol crystallization [16]. In parallel, elevated N-acetylarginine indicates arginine pathway disruption, whereby substrate consumption and NOS inhibition impair nitric oxide signaling and gallbladder contractility, leading to bile stasis [51]. Together, these findings highlight two complementary mechanisms—toxic bile acid accumulation and amino acid/NO pathway dysfunction—that converge to promote lithogenesis.

Correlation analyses further confirmed that protective taxa such as *Akkermansia muciniphila* and *CAG-448* were associated with lower levels of N-acetylarginine and GDCA, respectively, closely paralleling MR-based mediation effects. By validating these key metabolite shifts and their microbial associations, our results bridge genetic inference with experimental biology, thereby strengthening the evidence supporting a framework consistent with a causal link between diet, gut microbiota, and gallstone pathogenesis. However, the metabolic landscape extended well beyond these MR-concordant signatures. Our untargeted metabolomics revealed broader disturbances that provide additional mechanistic insights. Within the bile acid network, LD–induced mice exhibited a global shift toward a more hydrophobic pool, characterized by reduced primary bile acids, marked accumulation of secondary bile acids, and an elevated secondary/primary ratio. Concomitant increases in glycine- over taurine-conjugated bile acids, along with relative depletion of protective species such as ursodeoxycholic acid, further support the emergence of a lithogenic bile milieu [52]. In addition, metabolite class enrichment suggested parallel lipid alterations, including elevations in cholesterol and triglycerides, together with a decreased phosphatidylcholine/cholesterol ratio, consistent with impaired bile stability and enhanced crystallization risk [53].

The amino acid network and energy metabolism also displayed systemic perturbations. Beyond N-acetylarginine, we observed widespread elevations in N-acetylated amino acid species, accumulation of branched-chain amino acids, and activation of the tryptophan–kynurenine pathway. These changes point to global metabolic stress, insulin resistance, and low-grade inflammation, while the reduction in microbiota-derived indole derivatives suggests impaired gut barrier defense [54,55]. Finally, signatures of mitochondrial dysfunction were evident, as evidenced by increased long-chain acylcarnitine concentrations and a marked decline in the free carnitine/LCAC ratio, indicating disrupted β-oxidation and energy overload [56]. Consistent with this notion, recent evidence has shown that mitochondrial DNA variant 827A>G impairs respiratory chain function and activates AMPK-mediated cholesterol transport, thereby promoting gallstone formation in humans [57].

Collectively, these findings extend the MR-validated signatures of GDCA and N-acetylarginine into a broader network of bile acid imbalance, systemic amino acid stress, and mitochondrial energy failure, thereby providing a more comprehensive metabolic framework for gallstone pathogenesis.

The present study makes several contributions to the understanding of gallstone pathogenesis. First, by integrating MR with experimental validation, we provided convergent evidence supporting an association consistent with a causal link between diet-related microbial alterations, plasma metabolites, and gallstone risk, thereby complementing observational associations that are often subject to residual confounding and reverse causation. Second, our multi-omics strategy systematically connected gut microbial taxa, circulating metabolites, and lithogenesis, extending prior research that typically examined either microbiota or metabolic signatures in isolation. In particular, we identified candidate taxa such as *Akkermansia muciniphila* and *CAG-448* as potential microbial mediators, linking them to bile acid, amino acid, and energy metabolism in gallstone pathogenesis. Third, we uncovered additional pathways, including phospholipid remodeling and redox imbalance, that broaden the mechanistic framework beyond bile acid metabolism. Taken together, these findings advance current mechanistic understanding of gallstone disease and point to potential avenues for future investigation and intervention.

From a translational perspective, these findings underscore the gut microbiota–metabolite axis as a promising avenue for prevention and intervention in gallstone disease. Modifying dietary patterns—such as reducing pro-inflammatory load and increasing fiber intake—may beneficially reshape the gut microbiota and lower lithogenic metabolites, including GDCA and N-acetylarginine. Likewise, probiotic or prebiotic approaches, particularly those enhancing *Akkermansia muciniphila* or *CAG-448*, could strengthen protective metabolic pathways and help restore bile acid homeostasis, although such strategies will require validation in controlled trials. Preclinical studies have demonstrated that *A. muciniphila* and its derivatives exert protective effects across multiple disease models—for example, the tripeptide RKH mitigates lethal sepsis by blocking TLR4 signaling, and *A. muciniphila*-based interventions promote neurorepair via modulation of the gut–brain axis—thus supporting its translational potential while remaining at the preclinical stage of development [58,59]. In clinical translation, such dietary or microbial strategies are generally considered safe and feasible, but their optimal dosage, duration, and long-term efficacy in humans warrant further evaluation. The identified serum metabolites may also serve as minimally invasive biomarkers for early risk stratification, enabling precision surveillance in high-risk populations such as perimenopausal women or individuals with westernized dietary habits. Together, these translational implications highlight microbiota–metabolite interactions as a novel and actionable target for gallstone prevention and management.

This study encompasses certain restrictions that need recognition. First, MR analyses were based primarily on European cohorts, which may limit generalizability to other ancestries; replication in diverse populations is essential. Moreover, MR relies on key assumptions—such as sufficient instrument strength and no pleiotropy—the violation of which may bias causal inference. Second, some metabolites were instrumented by a limited number of genetic variants, raising the possibility of weak instrument bias, although F-statistic thresholds were applied to mitigate this concern. Third, animal validation was conducted in a short-term LD model, which may not fully capture the chronic and multifactorial nature of human gallstone disease. In addition, both MR and animal experiments cannot provide direct mechanistic proof, and the inferred causal relationships should, therefore, be interpreted with caution. Future studies should integrate bile, liver, and plasma metabolomics with longitudinal cohorts and interventional trials targeting diet, microbiota, or metabolic pathways to further clarify causality and enhance clinical applicability. Integration of host genetics, microbiome, and metabolomics in multi-ancestry cohorts will be particularly valuable for strengthening the evidence base.

## 5. Conclusions

In conclusion, by integrating global epidemiology, MR, and experimental validation, we provide evidence that diet-induced gut microbiota alterations are associated with gallstone formation through disruption of bile acid, amino acid, and lipid metabolism. Consistent risk metabolites—particularly GDCA and N-acetylarginine—emerged across genetic and experimental models, while untargeted metabolomics further revealed broader disturbances in bile acid balance, amino acid metabolism, and mitochondrial energy homeostasis. Microbial networks highlighted taxa such as *Akkermansia muciniphila* and *CAG-448* as critical mediators. Together, these findings not only advance mechanistic insight into the microbiota–metabolite axis of gallstone disease but also point to actionable dietary, microbial, and metabolic targets for prevention and intervention.

## Figures and Tables

**Table 1:** Data sources in this study.

	Ancestry	Participants	Consortium	GWAS ID	Ref
Gut microbiota (473)	European	5959	FINRISK	GCST90032172-GCST90032644	[23]
Plasma metabolites (1400)	European	8288	CLSA	GCST90199621-GCST90201020	[25]
Cholelithiasis	European	44,582 cases 397,583 controls	FinnGen R11	K11_CHOLELITH	[24]

**Table 2:** Study characteristics of four DII studies.

Study	Country	Source of Subjects	Morbidity Risk (%)		Odds Ratio (95% CI)
			High DII	Low DII	
Luo et al. [43]	USA	Cross-sectional + PSM	11.4	10.0	1.14 (1.01–1.29)
Jiang et al. [44]	USA	Cross-sectional	11.0	10.0	1.10 (1.01–1.19)
Wu et al. [7]	USA	Cross-sectional	14.4	10.0	1.44 (1.08–1.91)
Cheng et al. [45]	USA	Cross-sectional	15.2	10.0	1.52 (1.19–1.93)

High DII group and Low DII group were defined according to the median Dietary Inflammatory Index (DII) score in each study.

**Table 3:** Mediation effect of gut microbiota on the risk of cholelithiasis via metabolites.

Mediators	Gut Microbiota to Cholelithiasis					
	GM ^1^ to Mediator	Mediator to Cholelithiasis		Total Effect	Mediation Effect	
	GM	Beta1	Beta2		Effect	Proportion (95% CI)
N-acetylarginine	*Akkermansia muciniphila B*	0.072	0.12	−0.035	0.0087	25.10 (0–60.38)%
Glycodeoxycholate	*CAG-448 sp000433415*	−0.19	0.14	−0.098	−0.027	27.96 (0–63.11)%

^1^ GM, gut microbiota.

## Data Availability

This study analyzed global cholelithiasis burden using the 2021 Global Burden of Disease (GBD) dataset accessed via the Global Health Data Exchange (GHDx, Global Health Data Exchange). GWAS data involved in this article is available from OpenGWAS (https://gwas.mrcieu.ac.uk/ (accessed on 20 July 2025)), GWAS Catalog (https://www.ebi.ac.uk/gwas/ (accessed on 20 July 2025)) and FinnGen (https://www.finngen.fi/fi (accessed on 20 July 2025)) datasets. We list the detailed information of the GWAS datasets used in Table 1.

## Supplementary Materials

The following supporting information can be downloaded at: https://www.mdpi.com/article/10.3390/metabo15110714/s1, Table S1. Incidence of gallbladder and biliary diseases in 79 countries in 2021; Table S2. Causal effects between gut microbiota and cholelithiasis; Table S3. Reverse causal effects between gut microbiota and cholelithiasis; Table S4. Causal effects between plasma metabolites and cholelithiasis; Table S5. Reverse causal effects between plasma metabolites and cholelithiasis; Table S6. Causal effects between selected gut microbiota and plasma metabolites; Table S7. Mediating role of plasma metabolites in the gut microbiota–cholelithiasis association; Table S8. Gallbladder contractility parameters in control (CD) and lithogenic diet (LD) mice. Table S9. Gallbladder bile composition and cholesterol saturation index in CD and LD mice. Table S10. Quality control summary of shotgun metagenomic sequencing data from CD and LD mice.

## Abbreviations

GBD: Global Burden of Disease; DII: Dietary Inflammatory Index; GWAS: genome-wide association study; MR: mendelian randomization; SNP: single-nucleotide polymorphism; IV: instrumental variable; IVW: inverse variance weighting; WM: weighted median; MR-PRESSO: MR pleiotropy residual sum and outlier; LD: lithogenic diet; CD: chow diet; WGCNA: weighted gene co-expression network analysis; PCoA: principal coordinate analysis; ROS: reactive oxygen species; QC: quality control; CV: coefficient of variation; HMDB: Human Metabolome Database; KEGG: Kyoto Encyclopedia of Genes and Genomes; FDR: false discovery rate; OR: odds ratio; BCAA: branched-chain amino acids; LCAC: long-chain acylcarnitines; NOS: nitric oxide synthase; NO: nitric oxide; BA: Bile Acid; PL: Phospholipid; CHO: Cholesterol; CSI: Cholesterol Saturation Index; SPF: specific pathogen-free; LDA: Linear Discriminant Analysis; PERMANOVA: Permutational Multivariate Analysis of Variance; DDA: Data-Dependent Acquisition.
